# Test Anxiety and Coping Strategies Among Undergraduate Nursing Students: A Cross‐Sectional Study

**DOI:** 10.1002/nop2.70695

**Published:** 2026-07-14

**Authors:** Maria Elena Mariano, Nada Alatawi, Wejdan Albalawi, Afnan Alshehri, Ghada Alatawi, Aryam Altmimi, Danah Alshatb, Bushra Alshammari, Nabat Almalki, Mawahib Almalki

**Affiliations:** ^1^ Nursing Department Prince Sultan Military College of Health Sciences Dhahran Saudi Arabia; ^2^ Postnatal Unit King Salman Armed Forces Hospital Tabuk Saudi Arabia; ^3^ Prince Mansour Military Hospital Taif Saudi Arabia; ^4^ Neonatal Intensive Care Unit Armed Forces Hospital, King Abdul Aziz Naval Base Jubail Saudi Arabia; ^5^ Postnatal Unit Prince Sultan Military Medical City Riyadh Saudi Arabia; ^6^ Medical Surgical Nursing Department, College of Nursing University of Hail Hail Saudi Arabia; ^7^ School of Nursing and Midwifery Queen's University Belfast Belfast UK

## Abstract

**Aim:**

This study aimed to examine the level of test anxiety among nursing students in Saudi Arabia and to identify the coping strategies they use when facing pre‐exam anxiety and uncertainty.

**Methods:**

A cross‐sectional design was employed. Participants were recruited using convenience sampling. Data were collected from nursing students enrolled in regular and bridging programmes at a Saudi nursing college using a self‐administered questionnaire. Test anxiety was assessed using the Westside Test Anxiety Scale (WTAS), and coping strategies were measured using the Coping with Pre‐Exam Anxiety and Uncertainty (COPEAU) scale. Descriptive statistics and independent‐samples t‐tests were conducted to examine anxiety levels, coping patterns and differences between the regular and bridging programmes.

**Results:**

A total of 199 nursing students participated in the study. Overall, students demonstrated a moderate level of test anxiety, with cognitive worry, memory concerns and post‐exam performance anxiety emerging as the most prominent symptoms. Regarding coping strategies, students predominantly relied on task‐oriented and preparation‐focused approaches, reflecting strong engagement in planning, organisation and focused effort towards examinations. Seeking social support was also commonly utilised, indicating the importance of emotional and informational resources in managing exam‐related stress. In contrast, avoidance strategies were less strongly endorsed.

**Conclusion:**

Nursing students in Saudi Arabia experience moderate test anxiety but predominantly adopt adaptive coping strategies, particularly task‐oriented preparation and social support. These findings highlight the protective role of constructive coping behaviours in managing academic stress. Integrating structured exam preparation training, stress management programmes and accessible support services within nursing curricula is recommended to reduce cognitive worry and enhance students' academic resilience and psychological well‐being.

**Implications for Nursing Practice:**

These study findings can directly inform nursing education and student support. Embedding structured exam‐preparation and test‐taking skills, evidence‐based stress‐management and relaxation training and routine screening for elevated test anxiety within the curriculum may help reduce cognitive worry and memory‐related concerns. Peer‐support services, accessible counselling and academic advising, can reinforce the adaptive, task‐oriented and social‐support coping that students already favour and strengthen their academic resilience and psychological well‐being.

**Patient or Public Contribution:**

No patient or public contribution.

## 1 Introduction

Anxiety surrounding examinations is a persistent feature of higher education and is especially noticeable in nursing programmes that combine rigorous theoretical content with clinical assessments (Karaca et al. 2019). In nursing, students are expected to master complex knowledge and demonstrate competent practical performance. Thus, research has found that nursing students experience high levels of academic stress, likely due to the demanding and complex nature of nursing programmes (Nebhinani et al. 2020). Consequently, nursing students are recognised as prone to high stress; operating in high‐pressure evaluation environments, they are more vulnerable to test anxiety (Nebhinani et al. 2020). Test anxiety refers to physiological, phenomenological and behavioural responses associated with the fear of failing an examination or another assessment situation or the possibility of negative consequences (Zeidner 1998). Because nursing education relies heavily on high‐stakes examinations, the way students cope with this anxiety matters greatly: adaptive strategies such as problem‐solving and seeking social support are linked to lower stress, while maladaptive strategies like self‐criticism and social withdrawal are associated with higher stress and anxiety (Onieva‐Zafra et al. 2020). Although several interventions have shown promise, existing studies are limited by small samples and varied quality, so further investigation into test anxiety levels and effective coping strategies among nursing students is needed (Kaur Khaira et al. 2023; Quinn and Peters 2017). Beyond anxiety itself, students' coping mechanisms play a pivotal role in shaping academic outcomes (Labrague et al. 2017).

Loureiro et al. (2024) explored coping strategies and social support among nursing students, emphasising that these strategies are often discussed mainly in relation to stress management rather than academic stress. Despite growing interest in this area, few studies have concurrently reported the level of test anxiety and the specific coping strategies nursing students rely on during the high‐stakes pre‐examination period (Kaur Khaira et al. 2023; Onieva‐Zafra et al. 2020), and fewer still have done so within Saudi nursing programmes, where existing research has focused largely on general academic and clinical stress rather than test anxiety (El Sayed et al. 2024; Labrague, McEnroe‐Petitte, De Los Santos, and Edet 2018). This represents a knowledge gap, as understanding the extent of test anxiety and the coping strategies employed by students is essential for developing targeted, evidence‐based academic and psychological support interventions (Onieva‐Zafra et al. 2020).

Therefore, this study aims to examine the level of test anxiety among nursing students and to identify the coping strategies they use, in order to translate the quantitative findings into actionable recommendations for improving academic and psychological support services in Saudi nursing programmes.

## 2 Materials and Methods

### 2.1 Design

This study employed a cross‐sectional, correlational design. Data collection was conducted in March 2024 at the College of Health Sciences in Saudi Arabia. The study targeted nursing students enrolled across all academic levels.

### 2.2 Sample and Sampling

A convenience sampling approach was employed to recruit participants for this study. The sample consisted of female nursing students enrolled in the Bachelor of Science in Nursing (BSN) programme (regular and bridging). Participants were recruited based on accessibility and willingness to participate and were currently studying at the college.

### 2.3 Setting

The study was conducted at Health Sciences College in Saudi Arabia, a government‐affiliated institution providing undergraduate programmes in healthcare sciences in the Eastern Province of Saudi Arabia. The College offers a 4‐year BSN programme, including a 2‐year bridging track for diploma‐holding nurses. The programme includes frequent theoretical examinations and clinical evaluations, providing an appropriate setting for examining test anxiety levels and the coping strategies used by nursing students during the pre‐examination period.

### 2.4 Instruments

Data were collected using a structured self‐administered questionnaire comprising three sections. The first section captured sociodemographic and academic characteristics, including age group, marital status, academic track (regular or bridging) and current academic level. The second and third sections comprised the two standardised instruments described below. Both instruments are published, validated scales used with permission under their open/academic‐use terms and no modifications were made to the original items. Internal consistency in the present sample was acceptable for both scales (Cronbach's *α* > 0.70).

#### 2.4.1 Westside Test Anxiety Scale (WTAS)

Test anxiety was measured using the 10‐item WTAS. Items are rated on a 5‐point Likert scale ranging from 1 (*never or not at all true*) to 5 (*extremely or always true*). The total score was calculated by averaging the responses, yielding a final score ranging from 1.0 to 5.0, with higher scores indicating greater levels of test anxiety. Test anxiety levels were classified as follows: Low anxiety: 1.0–1.9, Normal anxiety: 2.0–2.5, High‐normal anxiety: 2.6–2.9, Moderately high anxiety: 3.0–3.4, High anxiety: 3.5–3.9, Extremely high anxiety: 4.0–5.0 (Driscoll 2007). The WTAS is a validated measure of test anxiety with reported good internal consistency (Cronbach's *α* = 0.78), and its construct validity has been supported by its association with examination performance (Driscoll 2007). A higher mean score reflects a higher level of test anxiety, interpreted according to the six classification bands listed above.

#### 2.4.2 Coping With Pre‐Exam Anxiety and Uncertainty (COPEAU)

Coping strategies were assessed using the COPEAU scale. The instrument consists of 21 items divided into three subscales (7 items each): (1) task‐orientation and preparation, (2) seeking social support and (3) avoidance. Each item is rated on a 6‐point Likert scale ranging from 1 (*definitely not true*) to 6 (*definitely true*). Higher scores indicate greater use of the respective coping strategy (Stöber 2004).

### 2.5 Recruitment Procedures

Data were collected using a structured online questionnaire administered through a secure electronic platform. Participation was voluntary, and responses were kept anonymous to ensure confidentiality. The survey included a brief introduction explaining the purpose of the study and emphasising voluntary participation; completion and submission of the questionnaire were taken as implied consent. Because the survey was anonymous and completed in a single session, participants were free to decline participation or to discontinue at any point before submitting their responses. However, once a response was submitted it could not be identified or withdrawn, given the anonymous, single‐submission cross‐sectional design. During the data collection period, the questionnaire was distributed to all eligible nursing students (*N* = 199), and all students completed the survey, resulting in a 100% response rate.

Completion of the survey required approximately 15–20 min. Ethical approval for the conduct of the study was granted by the Institutional Review Board (IRB) of Prince Sultan Military College of Health Sciences (IRB approval number: IRB‐2024‐NUR‐10).

### 2.6 Statistical Analysis

Data were analysed using the Statistical Package for the Social Sciences (SPSS). Descriptive statistics, including frequencies and percentages, were used to report demographic characteristics, while means and standard deviations were used for levels of test anxiety and coping strategies. A *p* value of ≤ 0.05 was considered statistically significant. Independent‐samples *t*‐tests were also performed to compare differences between regular BSN students and bridging students.

## 3 Results

A total of 199 nursing students participated in the survey. The demographic analysis showed that most participants were aged 21–23 years (32.2%). The majority of students were single (80.9%), while 17.6% were married, and only a small proportion were widowed (0.5%) or divorced (1.0%). Regarding academic track, 59.3% were regular students, whereas 40.7% were bridging students. In terms of academic level, the highest proportion of students were in Level 4 (30.7%), followed by Bridging L2 (23.6%), Bridging L4 (17.1%), Regular L8 (15.6%) and Regular L6 (13.1%), Table 1.

**TABLE 1:** Demographic data.

Variable	Category	Frequency (*n*)	Percentage (%)
Age group	18–20	56	28.1
	21–23	64	32.2
	24–26	31	15.6
	≥ 27	48	24.1
Marital status	Single	161	80.9
	Married	35	17.6
	Widowed	1	0.5
	Divorced	2	1.0
Academic track	Regular	118	59.3
	Bridging	81	40.7
Current academic level	Level 4	61	30.7
	Level 6	26	13.1
	Level 8	31	15.6
	Bridging Level 2	47	23.6
	Bridging Level 4	34	17.1

Table 2 presents participants' responses to the (WTAS). The WTAS overall mean = 3.01, SD = 1.02. Nursing students demonstrated a moderate level of test anxiety, with item means ranging from 2.78 to 3.34. The highest mean score was observed for *worry about not remembering the material during the exam* (*M* = 3.34, SD = 1.28), indicating that cognitive worry and memory concerns were the most prominent anxiety symptoms. Similarly, participants reported substantial *post‐exam worry about performance* (*M* = 3.25, SD = 1.31), reflecting persistent evaluative concerns beyond the testing situation.

**TABLE 2:** Participants' responses to Westside Test Anxiety Scale (*N* = 199).

WTAS item	Not at all *n* (%)	Slightly *n* (%)	Moderately *n* (%)	Highly *n* (%)	Extremely *n* (%)	Mean	SD
The closer I am to a major exam, the harder it is for me to concentrate on the material	25 (12.6)	39 (19.6)	65 (32.7)	38 (19.1)	32 (16.1)	3.07	1.24
When I study, I worry that I will not remember the material on the exam	14 (7.0)	45 (22.6)	53 (26.6)	33 (16.6)	54 (27.1)	3.34	1.28
During important exams, I think that I am doing awful or that I may fail	45 (22.6)	38 (19.1)	57 (28.6)	33 (16.6)	26 (13.1)	2.78	1.32
I lose focus on important exams, and I cannot remember material that I knew before the exam	30 (15.1)	51 (25.6)	61 (30.7)	28 (14.1)	29 (14.6)	2.87	1.26
I finally remember the answers to exam questions after the exam is already over	24 (12.1)	51 (25.6)	51 (25.6)	42 (21.1)	31 (15.6)	3.03	1.26
I worry so much before a major exam that I am too worn out to do my best on the exam	27 (13.6)	47 (23.6)	50 (25.1)	39 (19.6)	36 (18.1)	3.05	1.31
I feel out of sorts or not really myself when I take important exams	42 (21.1)	40 (20.1)	50 (25.1)	34 (17.1)	33 (16.6)	2.88	1.37
I find that my mind sometimes wanders when I am taking important exams	30 (15.1)	49 (24.6)	48 (24.1)	45 (22.6)	27 (13.6)	2.95	1.27
After an exam, I worry about whether I did well enough	26 (13.1)	30 (15.1)	54 (27.1)	46 (23.1)	43 (21.6)	3.25	1.31
I struggle with writing assignments, or avoid them as long as I can. I feel that whatever I do will not be good enough	42 (21.1)	39 (19.6)	48 (24.1)	39 (19.6)	31 (15.6)	2.89	1.36

Moderate levels of anxiety were also evident in items related to *concentration difficulties* as exams approached (*M* = 3.07, SD = 1.24), *physical and mental exhaustion before major exams* (*M* = 3.05, SD = 1.31) and *remembering answers only after the exam had ended* (*M* = 3.03, SD = 1.26). In contrast, comparatively lower mean scores were reported for *fear of failing during the exam* (*M* = 2.78, SD = 1.32) and *feeling out of sorts during important exams* (*M* = 2.88, SD = 1.37), suggesting that emotional distress was less pronounced than cognitive worry.

The overall mean of COPEAU task‐orientation (*M* = 4.19; SD = 1.11). The highest reported coping strategy was task‐orientation/preparation (*M* = 4.19), indicating that students primarily relied on planning, organising and concentrating their efforts to manage pre‐exam anxiety. This was followed by seeking social support (*M* = 4.00), reflecting frequent use of emotional and informational support from others. The lowest mean score was observed for avoidance strategies (*M* = 3.81), suggesting that distraction and disengagement were less commonly used compared with more adaptive coping approaches. Table 3 shows that, overall, participants reported high levels of task‐oriented and preparation‐focused coping strategies. Mean scores across items ranged from 3.98 to 4.42, indicating that most students tended to agree or strongly agree with behaviours reflecting structured preparation, prioritisation and focused effort towards examinations. The highest mean score was observed for ‘I think about how I can best prepare for the exam’ (*M* = 4.42), followed closely by ‘I concentrate all my efforts on the exam’ (*M* = 4.40), suggesting strong cognitive and behavioural engagement in exam preparation. Even the lowest‐scoring item, ‘I do what needs to be done, one step at a time’ (*M* = 3.98), remained close to the agreement range. Collectively, these findings indicate that the majority of students rely on adaptive, task‐oriented coping strategies when facing examinations.

**TABLE 3:** COPEAU‐task‐orientation/preparation (*N* = 199).

Item	Definitely not *n* (%)	Mostly not *n* (%)	Slightly not *n* (%)	Slightly *n* (%)	Mostly *n* (%)	Definitely *n* (%)	Mean
I think about how I can best prepare for the exam	15 (7.5)	12 (6.0)	18 (9.0)	37 (18.6)	63 (31.7)	54 (27.1)	4.42
I concentrate on how I'm going to deal with the exam and, if necessary, let other things slide	14 (7.0)	17 (8.5)	32 (16.1)	53 (26.6)	48 (24.1)	35 (17.6)	4.05
I cut back on my leisure time to prepare for the exam	16 (8.0)	19 (9.5)	24 (12.1)	51 (25.6)	46 (23.1)	43 (21.6)	4.11
I take extra time to prepare for the exam	14 (7.0)	22 (11.1)	29 (14.6)	46 (23.1)	42 (21.1)	46 (23.1)	4.09
I do what needs to be done, one step at a time	12 (6.0)	27 (13.6)	31 (15.6)	52 (26.1)	37 (18.6)	40 (20.1)	3.98
I put other activities to one side and concentrate on the exam coming up	10 (5.0)	18 (9.0)	27 (13.6)	42 (21.1)	60 (30.2)	42 (21.1)	4.26
I concentrate all my efforts on the exam	7 (3.5)	15 (7.5)	23 (11.6)	46 (23.1)	63 (31.7)	45 (22.6)	4.40

The overall mean of COPEAU seeking social support (*M* = 4.00; SD = 1.22). Table 4 shows that, overall, nursing students demonstrated moderate to high use of seeking social support coping strategies, with item means ranging from 3.84 to 4.16. The highest mean score was observed for *talking to others to find out more about the exam* (*M* = 4.16), followed by *talking to someone about how I feel* (*M* = 4.13), indicating a strong tendency towards informational and emotional support‐seeking. In contrast, *seeking sympathy and understanding from others* showed the lowest mean (*M* = 3.84), suggesting comparatively less reliance on passive emotional reassurance. Collectively, the distribution of responses, with a predominance of ‘slightly’, ‘mostly’ and ‘definitely’ endorsement categories, reflects that social support is a commonly utilised coping approach among the participants.

**TABLE 4:** COPEAU‐seeking social support.

Item	Definitely not *n* (%)	Mostly not *n* (%)	Slightly not *n* (%)	Slightly *n* (%)	Mostly *n* (%)	Definitely *n* (%)	Mean
I ask people who have had similar experiences what they did/would do	21 (10.6)	21 (10.6)	19 (9.5)	61 (30.7)	38 (19.1)	39 (19.6)	3.96
I discuss my feelings with someone	13 (6.5)	26 (13.1)	27 (13.6)	52 (26.1)	42 (21.1)	39 (19.6)	4.01
I try to get advice from someone about what to do	14 (7.0)	28 (14.1)	30 (15.1)	53 (26.6)	46 (23.1)	28 (14.1)	3.87
I attempt to get the emotional support of friends or relatives	16 (8.0)	20 (10.1)	19 (9.5)	66 (33.2)	43 (21.6)	35 (17.6)	4.03
I try to get sympathy and understanding from others	21 (10.6)	25 (12.6)	26 (13.1)	52 (26.1)	43 (21.6)	32 (16.1)	3.84
I talk to someone about how I feel	15 (7.5)	23 (11.6)	21 (10.6)	49 (24.6)	44 (22.1)	47 (23.6)	4.13
I talk to others to find out more about the exam	17 (8.5)	13 (6.5)	22 (11.1)	53 (26.6)	58 (29.1)	36 (18.1)	4.16

However, COPEAU Avoidance mean (*M* = 3.81; SD = 1.24). Table 5 shows moderate use of avoidance coping strategies among nursing students, with mean scores ranging from 3.64 to 4.13. The highest endorsement was observed for *convincing oneself that the situation is not entirely negative* (*M* = 4.13), suggesting the use of cognitive reframing as a primary avoidance‐related response. In contrast, behaviours reflecting active disengagement, such as *making a conscious effort to think about something else* (*M* = 3.64) and *persuading oneself not to care about the exam* (*M* = 3.66), showed lower mean scores. Overall, the distribution of responses indicates that while avoidance strategies are present, they are generally less strongly endorsed than problem‐focused or social support coping approaches, reflecting a balanced but cautious reliance on avoidance when managing exam‐related stress.

**TABLE 5:** COPEAU—avoidance.

Item	Definitely not *n* (%)	Mostly not *n* (%)	Slightly not *n* (%)	Slightly *n* (%)	Mostly *n* (%)	Definitely *n* (%)	Mean
I convince myself that it's not all bad	15 (7.5)	19 (9.5)	25 (12.6)	48 (24.1)	50 (25.1)	42 (21.1)	4.13
I put thoughts of the exam out of my mind	21 (10.6)	21 (10.6)	27 (13.6)	58 (29.1)	38 (19.1)	34 (17.1)	3.87
I try not to think about the exam	25 (12.6)	24 (12.1)	29 (14.6)	46 (23.1)	41 (20.6)	34 (17.1)	3.78
I turn to other activities for diversion	12 (6.0)	25 (12.6)	37 (18.6)	54 (27.1)	44 (22.1)	27 (13.6)	3.87
I persuade myself that I don't care about the exam	29 (14.6)	28 (14.1)	26 (13.1)	49 (24.6)	32 (16.1)	35 (17.6)	3.66
I go to the movies or watch TV so I don't think about the exam so much	36 (18.1)	22 (11.1)	21 (10.6)	38 (19.1)	49 (24.6)	33 (16.6)	3.71
I make a conscious effort to think about something else	24 (12.1)	28 (14.1)	29 (14.6)	55 (27.6)	41 (20.6)	22 (11.1)	3.64

Independent‐samples *t*‐tests revealed no significant differences between regular BSN and Bridging students in test anxiety or coping strategies. Both groups demonstrated comparable levels of test anxiety and similar patterns of task‐oriented, social support and avoidance coping. These findings suggest that programme type did not influence anxiety levels or coping behaviours among the studied nursing students (Table 6).

**TABLE 6:** Comparison of regular and bridging students.

Variable	Regular (*N* = 118) mean	Regular SD	Bridging (*N* = 81) mean	Bridging SD	*p*
WTAS (1.0–5.0)	3.02	1.03	3.00	1.02	0.89
COPEAU task‐orientation (1–6)	4.19	1.12	4.18	1.09	0.92
COPEAU social support (1–6)	3.99	1.20	4.01	1.25	0.90
COPEAU avoidance (1–6)	3.90	1.22	3.68	1.28	0.21

*Note:* Independent‐samples *t*‐tests were used. No statistically significant differences were observed between groups.

## 4 Discussion

This study shows that nursing students experience moderate test anxiety, mainly driven by cognitive concerns such as memory, concentration and post‐exam evaluation rather than emotional distress. Consistent with these findings, a study conducted in Saudi universities found that a moderate level of anxiety may serve as a motivational factor that enhances students' effort and preparation (Dawood et al. 2016). This result may reflect the motivating effect of moderate anxiety, especially during critical academic stages, which can increase students' motivation to prepare for exams, leading to better performance outcomes (Balogun et al. 2017). According to Ahmetovic et al. (2020) the relationship between anxiety and motivation is not linear but dynamic. While higher levels of anxiety tend to decrease intrinsic motivation and increase reliance on extrinsic motives such as fear of failure or external expectations, a moderate level of anxiety can serve a facilitative role by enhancing students' alertness and motivation to perform better. This suggests that anxiety, when maintained at an optimal level, may act as a motivating force rather than a purely detrimental factor in academic achievement. On the other hand, Balogun et al. (2017) found that test anxiety negatively affects academic performance; however, this effect was moderated by achievement motivation.

However, Dawood et al. (2016) reported that test anxiety is common among nursing students, with more than half experiencing moderate levels and around 14% experiencing severe anxiety. Further Farrag et al. (2020) found that 68.1% of students experienced high test anxiety. Although previous studies conducted within the region have reported varying levels of test anxiety among students in health‐related fields, these differences do not consistently translate into academic performance outcomes (Dawood et al. 2016; Farrag et al. 2020). More recent evidence is consistent with these observations: a systematic review and subsequent cross‐sectional and review studies continue to report moderate‐to‐high test anxiety among nursing students and underscore the value of structured, skills‐based interventions for reducing it (Kaur Khaira et al. 2023, 2024; McCormick and Lamberson 2024). Comparable patterns of academic stress and psychological distress have also been documented among Saudi nursing students (Andargeery et al. 2024).

The present study also explored the most commonly used coping strategies among nursing students prior to examinations. Among the three measured domains, task‐oriented coping emerged as the most frequently utilised strategy. Similar findings were reported by Onieva‐Zafra et al. (2020) who noted that nursing students frequently employed task‐oriented coping, particularly in higher academic levels. This type of coping may reflect a positive adaptive behaviour in response to academic demands. Conceptually, task‐oriented coping corresponds to problem‐focused coping in the Lazarus and Folkman (1984) transactional framework, in that its primary function is to manage or alter the stressor itself, here, the examination, through planning, preparation and focused effort, rather than to directly regulate emotional states. Accordingly, such strategies may serve a protective function indirectly: by enabling students to act on and reduce the source of stress, effective preparation can lower perceived threat and, in turn, support emotional stability and academic performance. As a result, task‐oriented (problem‐focused) coping has been widely recognised in the literature as an effective and adaptive method of managing academic challenges in stressful environments such as nursing education (Quinn and Peters 2017). Seeking social support also emerges as a commonly used strategy, reflecting the importance of both informational and emotional resources in managing academic stress. Consistent with the global literature, studies consistently argue that seeking social support constitutes a core coping strategy for nursing students facing academic stress. Evidence by Onieva‐Zafra et al. (2020) emphasises that social support, along with problem‐solving, is used to cope with perceived stress, highlighting the protective role of supportive relationships in reducing anxiety and improving academic experiences. In addition, Labrague, McEnroe‐Petitte, Papathanasiou, et al. (2018) argue that social support is particularly effective because it provides both informational guidance and emotional reassurance, enabling students to regulate exam‐related stress and mitigate anxiety more effectively. Further supporting this argument, Almeida et al. (2018) highlight that nursing students who report stronger and more satisfying support from family and friends experience lower levels of academic stress, especially in theoretical components, underscoring the central role of support networks in fostering psychological resilience within academic environments.

Nevertheless, it is worth noting that avoidance strategies were also present among students, though to a lesser extent. Based on these results, Quinn and Peters (2017) argued that students who relied on avoidance coping were less equipped to manage exam‐related pressures effectively. However, mild avoidance can serve as a buffer against overwhelming stress. For instance, anticipatory coping, where individuals prepare for potential stressors while managing their worries, can enhance overall preparedness (Aspinwall et al. 2005). The absence of statistically significant differences between regular BSN and bridging students indicates that programme type was not associated with differences in test anxiety or coping strategy use. Overall, students from both academic tracks reported comparable levels of test anxiety and similar patterns of task‐oriented, social support and avoidance coping. However, further longitudinal research is needed to determine whether these strategies lead to long‐term academic benefits or simply reflect immediate stress management behaviours.

### 4.1 Strengths and Limitations

This study provides a comprehensive assessment of test anxiety and coping strategies among undergraduate nursing students using two validated instruments, the WTAS and the COPEAU. The use of standardised, psychometrically sound measures enhances the reliability and credibility of the findings.

However, this study has several limitations. Convenience sampling from a single institution and the inclusion of only female nursing students limit the representativeness and generalisability of the findings (Albikawi 2022; Alasqah et al. 2024). Reliance on self‐report questionnaires may have introduced social desirability and recall biases, and participants' responses may not fully reflect their actual coping behaviours or academic performance. Additionally, the cross‐sectional design precludes causal inferences and does not capture changes in test anxiety or coping strategies over time. Finally, subgroup analyses by academic level were based on relatively small sample sizes, limiting statistical power and increasing the uncertainty of these findings; therefore, these results should be interpreted with caution. Future research should employ multicentre sampling, include both male and female nursing students, adopt longitudinal designs and incorporate objective measures of academic performance to strengthen the generalisability and robustness of the evidence.

### 4.2 Future Directions

Future research should adopt longitudinal designs to track changes in test anxiety and coping across academic progression and key transition points. Expanding samples across multiple institutions would enhance external validity.

### 4.3 Implications for Nursing Practice and Education

The findings offer several practical steps for nurse educators and programme leaders. First, because students' anxiety was driven mainly by cognitive worry, memory and concentration concerns rather than emotional distress, faculty should embed structured test‐taking and exam‐preparation skills directly into course delivery and assessment preparation. Second, short stress‐management tools such as cognitive‐behavioural techniques, mindfulness and breathing exercises can be integrated into pre‐examination periods to reduce cognitive interference. Third, since students relied heavily on problem‐solving and social support to cope, programmes should strengthen these habits through peer study groups, mentoring by senior students and faculty‐led review sessions, while making counselling and academic support easy to find and use. Finally, academic advisors should proactively address workload planning, assessment timelines and coping resources during advising sessions, so that support is offered before anxiety escalates.

## 5 Conclusion

This study provides a comprehensive overview of test anxiety and coping strategies among nursing students in Saudi Arabia, revealing a predominantly moderate level of test anxiety characterised mainly by cognitive worry, concentration difficulties and post‐exam evaluative concerns. Anxiety symptoms were more strongly related to worries about memory and performance rather than emotional distress, indicating that cognitive aspects of anxiety constitute the primary challenge faced by students during examinations.

In response to exam‐related stress, students demonstrated a clear preference for adaptive coping strategies, particularly task‐oriented and preparation‐focused behaviours, reflecting strong engagement in planning, organisation and focused effort. Seeking social support also emerged as a commonly utilised approach, highlighting the importance of peer and interpersonal resources in managing academic stress. In contrast, avoidance strategies were less prominently endorsed, indicating that students generally relied more on constructive and problem‐focused coping rather than disengagement.

Collectively, these findings suggest that while test anxiety remains a prevalent concern among nursing students, the widespread use of adaptive coping strategies may serve as a protective factor that supports academic functioning. The results underscore the importance of integrating structured preparation skills, stress management training and accessible support systems within nursing programmes to enhance students' psychological well‐being and academic resilience.

## Author Contributions

M.E.M., N.A., W.A., A.A., G.A., A.A. and D.A. contributed to conceptualising and designing the study and collecting the data. M.E.M, N.A., W.A., A.A., G.A., A.A., D.A., B.A., N.A. and M.A. conducted and rechecked the statistical analysis and interpretation. M.E.M, B.A., N.A. and M.A. drafted the original manuscript. All authors critically reviewed and revised the manuscript and approved the final version for submission.

## Funding

The authors have nothing to report.

## Disclosure

No artificial intelligence (AI) tools or writing assistance were used in the preparation of this manuscript.

## Conflicts of Interest

The authors declare no conflicts of interest.

## Data Availability

All data are included in this published article.
